# Drug Interaction between Dexamethasone and Ketoprofen with Thiopental in Male Dogs: Effect on the Recovery from Anesthesia and Pharmacokinetics Parameters

**DOI:** 10.1155/2022/3016853

**Published:** 2022-01-18

**Authors:** Mahdieh Raeeszadeh, Mohammad Pouya Ghaffari

**Affiliations:** ^1^Department of Basic Sciences, Sanandaj Branch, Islamic Azad University, Sanandaj, Iran; ^2^Graduate of Faculty of Veterinary Sciences, Sanandaj Branch, Islamic Azad University, Sanandaj, Iran

## Abstract

Drug-drug interactions (DDIs) are an important part of clinical veterinary pharmacology. Forty-two healthy mixed breed male dogs were randomly divided into three groups. The control group (C) received normal saline (1 mg/kg) 5 minutes before intravenous administration of thiopental (17 mg/kg), the T1 group received ketoprofen (2.2 mg/kg), and the T2 group received dexamethasone (0.2 mg/kg) 5 minutes before thiopental, respectively. Clinical parameters of anesthesia, heart rate, respiration rate, and electrocardiography were measured. Serum samples were also used to assay thiopental concentration using the high-performance liquid chromatography (HPLC) method, and then, thiopental pharmacokinetic parameters were calculated. Changes in the heart rate and respiration were significant intragroup differences 5 and 10 minutes after anesthesia, respectively. Recovery time parameters showed a significant increase between T1 and control groups (*P* < 0.05). Elimination rate and half-life of thiopental in the T1 group compared to the control and T2 groups showed a significant decrease and increase, respectively. In addition, the distribution of thiopental in T1 showed a significant increase compared to other groups. However, thiopental clearance in T1 and T2 groups had no significant difference with control (*P* > 0.05). It can be concluded that drug interaction between ketoprofen and thiopental causes to change in the pharmacokinetics parameters and recovery time from anesthesia in comparison with dexamethasone.

## 1. Introduction

Since the advent of the science of surgery, the use of safe methods to reduce pain and complications of surgery has been considered. Balanced and safe anesthesia is one of the most important needs of a successful and uncomplicated surgery [1]. Anesthesia is performed by various methods such as injection and inhalation methods. The choice of anesthesia depends on a wide range of factors, including the patient's condition and the type of surgery performed by the surgeon. Despite the widespread use of inhaled anesthesia in veterinary medicine and the helpful benefits of its use, the use of injectable anesthesia is still necessary [2, 3]. In some cases, the use of injectable anesthesia increases the induction and depth of anesthesia, which can be effective in emergency anesthesia and field surgery [3]. In addition, so far, no anesthetic has been provided alone that can provide all aspects of ideal and balanced anesthesia without any side effects, and each patient with special physiological and pathological conditions needs a special anesthesia diet and special care [4, 5]. Therefore, in most cases, to minimize the side effects of anesthetics and establish stability in the patient's vital parameters, a combination of anesthesia methods and a combination of complementary drugs with anesthetics are used [6]. The concomitant use of multiple drugs can target several therapeutic goals or can focus a standard action upon one single purpose or disease [7].

The positive effects of drug interactions include reducing the required dose of the drug while producing the same effect and reducing its toxicity and the incidence and extent of side effects, minimizing the rate of drug resistance and making a selective and synergistic effect on the target [8]. However, drug-drug interactions (DDIs) are a part of clinical pharmacology that investigates the risk of drug interactions to predict the consequences of combined drug use. It can alter pharmacokinetic parameters and thus drug performance [8, 9]. These events are of interest to many researchers and physicians [10, 11]. DDI is more likely to occur due to taking different medications during anesthesia [8].

Barbiturates are a group of sedatives used to treat seizures, insomnia, and anxiety, and induce anesthesia. Sodium thiopental sodium as a very fast-acting barbiturate is mainly used with analgesics or muscle relaxants to induce general anesthesia in short-term surgery with minimal painful stimulation [12, 13]. Thiopental is a very rapid depressant that has distribution into the central nervous system within 30–45 seconds and, although it has no role in inducing analgesia, causes hypnosis and anesthesia. This feature has led to its widespread use in veterinary medicine to induce anesthesia in animals. Thiopental is commonly used in surgery to induce anesthesia, and its use is not recommended to maintain anesthesia, because it displays zero-order elimination pharmacokinetics [14]. As mentioned earlier, one of the limitations of using thiopental in anesthesia, especially for orthopedic surgeries, is the lack of analgesic properties. In this case, using nonsteroidal analgesic and anti-inflammatory agents such as ketoprofen or dexamethasone is recommended. Therefore, it is usually used in combination with other sedatives and analgesics. Ketoprofen is a propionic acid derivative and a nonsteroidal anti-inflammatory drug (NSAID) used in combination with thiopental. Its anti-inflammatory effect is weaker than other NSAIDs [15]. In addition to ketoprofen, dexamethasone can also be used with thiopental to induce general anesthesia [16, 17]. Dexamethasone exerts widely anti-inflammatory effects by preventing the accumulation of inflammatory cells in the area of inflammation, inhibiting phagocytosis and releasing enzymes responsible for inflammation, inhibiting the production and release of chemical mediators of inflammation [17, 18]. Dexamethasone has been used widely in clinical specialties, including anesthesia. It is regarded as one of the ideal perioperative agents, being readily available, cheap, anti-inflammatory agents. It prevents and treats postoperative nausea and vomiting (PONV), promotes appetite, and is a good analgesic agent either intravenously or as an adjuvant to peripheral nerve blocks. It provides a sense of well-being and is considered a good quality of recovery and early discharge in patients from anesthesia [19]. However, concomitant use of thiopental with analgesics and anti-inflammatory drugs such as dexamethasone and ketoprofen may be associated with drug interactions. Hence, it seems necessary to study their effect on clinical conditions and pharmacokinetic parameters of thiopental. Therefore, this study aimed to evaluate the drug interaction of thiopental with dexamethasone and ketoprofen on thiopental pharmacokinetic parameters and some critical parameters during anesthesia in the male dogs.

## 2. Materials and Methods

### 2.1. Animals

All stages of this study under the “Guidelines for the care and use of research animals” approved by the Ethics Committee of Kurdistan University of Medical Sciences with the code IR.MUK. REC. 1399.156 were performed. In a randomized controlled clinical intervention, forty-two healthy mixed breed male dogs 1–1.5 years old (23 ± 2.2 kg) selected from the animal shelter at the Faculty of Veterinary Medicine of Sanandaj Azad University were used in this study. The animals were kept in the animal shelter and had free access to water and dog food.

### 2.2. Preanesthetic Evaluation of Blood and Serum Parameters

One week after adaptation to the experimental conditions, after the initial physical examination, to confirm the health of the animals, blood samples were taken from the cephalic vein to evaluate hematological and biochemical parameters one day before the study. After separation of serum by centrifugation (3000 rpm, 10 min), hematological parameters such as mean corpuscular volume (MCV), cell blood count (CBC), hemoglobin (HB), hematocrit (HCT), mean corpuscular hemoglobin concentration (MCHC), and biochemical parameters including aspartate transaminase (AST), alanine transaminase (ALT), total protein, albumin, urea, and creatinine were measured using standard methods to confirm the health of the animals. We did not have fluid therapy in the animals used.

### 2.3. Study Design

After confirmation of their health, the dogs were randomly divided into three equal groups of fourteen as follows:

  Control group (C): animals received normal saline (1 mg/kg) 5 minutes before administration of sodium thiopental (17.5 mg/kg).
  Experiment group 1 (T1): animals received ketoprofen (2.2 mg/kg) 5 minutes before sodium thiopental administration.
  Experimental group 2 (T2): animals received dexamethasone (0.1 mg/kg) 5 minutes before sodium thiopental administration.

Thiopental sodium (0.5 g vials; Sandoz, Austria, Lot No. 150566), ketoprofen (Ketofen 10%, Abureyhan, Tehran, Iran), dexamethasone (Dexa Vet 0.2%, Abureyhan, Tehran, Iran), and normal saline (sodium chloride 0.9%, Martyr Judge Serum Company, Tabriz, Iran) were purchased from local suppliers.

### 2.4. Monitoring and Anesthetic Procedure and Evaluation of Recovery Time

An intravenous catheter was inserted into the cephalic vein to inject saline and drugs, and collect blood samples to evaluate pharmacokinetic parameters of thiopental. Heart rate and respiration were measured at consecutive times after induction of anesthesia. Before and after induction of thiopental anesthesia and during recovery, ECG was obtained from animals in different groups. ECG analysis and interpretation were performed by a cardiologist. Then, blood samples were taken at 0, 5, 10, 20, 30, 45, 60, 90, and 120 hours and serum were separated (3000 rpm, 10 min) to measure thiopental concentration. The concentration of thiopental in serum was measured using high-performance liquid chromatography (HPLC) to draw the concentration diagram in time. Thiopental pharmacokinetic parameters such as half-life, K elimination, the volume of distribution, and clearance were then calculated in different groups [20]. Recovery times (minute), including return palpebral reflex, sitting position, trying to stand, imbalanced walking, and normal walking, were assessed in all groups [21].

### 2.5. Electrocardiographic Analysis

The ECG record was taken with a paper speed of 50 mm/sec at normal filtering. ECG parameters were measured manually. QT interval was calculated from the lead II using calipers. QT interval was defined as the interval between the beginning of the QRS complex and the end of the *T* wave. Three consecutive cycles were measured in each of standard lead II, and a mean value was calculated from the three values [22].

### 2.6. Measurement of Thiopental Concentration by HPLC Method

The standard stock solution was prepared by dissolving 800 mg of thiopental in 100 ml of methanol. This solution was diluted with HPLC grade water (1 : 1) to precipitate the protein. 100 *μ*l of filtered plasma was mixed with 200 *μ*l of protein precipitating solution (100 ml of methanol with 0.5 g of zinc sulfate and 1 ml of ethylene glycol) for 10 seconds. Then, 200 *μ*l of 10% trichloroacetic acid solution was added. The solution is then remixed for 20 seconds and centrifuged at 4500 g for 5 minutes. After centrifugation, 20 *μ*l of supernatant was prepared for injection in the HPLC column. Acetonitrile was measured at 0.8 ml/min and acetic acid at 1% as the mobile phase at a flow rate of 1.2 ml/min with a wavelength of 260 nm [20].

### 2.7. Measurement of Pharmacokinetic Parameters

Pharmacokinetic parameters were analyzed by a noncompartment model and calculated using formulas related to half-life (*t*_1/2_), the volume of distribution (*Vd*), and clearance (*Cl*) [23, 24].

(1) $$\begin{array}{c} t_{1/2}=\frac{0.7}{k_{el}},\\ Vd=\frac{\text{Dose}}{C0},\\ Cl=Vd\times k_{el}. \end{array}$$

### 2.8. Data Analysis

Data were reported as mean ± standard error of the mean (mean ± SEM). The normality of the data was checked by default tests such as Kolmogorov–Smirnov and equality test, and the amount of sig was more than 0.05. Therefore, recovery time and pharmacokinetic parameters of thiopental data were analyzed by one-way analysis of variance (ANOVA). Repeated measures ANOVA was also used to analyze the data from heart rate and respiratory rate at the desired times among groups. The mean of data between the groups was compared by Tukey's post hoc test. All statistical analyses of the data were performed by SPSS (version 23), and *P* < 0.05 was considered as a significant level.

## 3. Results

Hematological and biochemical parameters in this study were measured to evaluate the health status of the animals used in this study. The results of this evaluation showed that these parameters were typical in the study groups. No significant differences were observed in the biochemical and hematological parameters among groups (*P* < 0.05) (Table 1).

**Figure 1:**
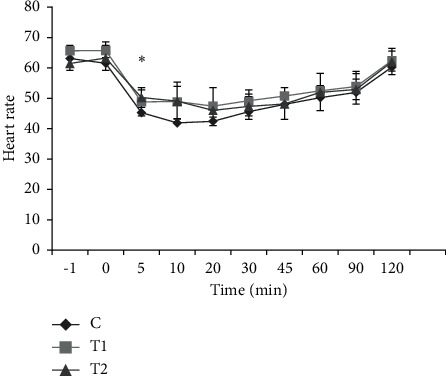
Changes in heart rate in the studied groups before and after anesthesia. All values are presented as the mean ± standard error of the mean (mean ± SEM). ^*∗*^is a significant difference within the intragroup. C: control group. T1: treatment group 1. T2: treatment group 2.

Analysis of the results showed that from 5 minutes after injection to 45 minutes after that, heart rate showed a significant difference compared to before injection and at the time of thiopental injection. However, these changes did not show a significant difference in comparison between groups (Figure 1).

**Figure 2:**
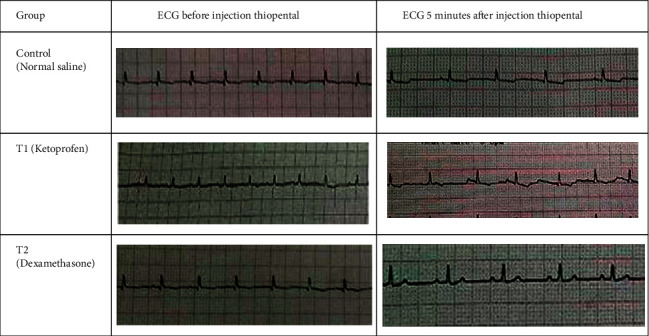
A typical sample of electrocardiogram (ECG) taken from animals in control and treatment groups before and 5 minutes after thiopental injection.

To monitor changes in heart rate and possible arrhythmias, all animals before, at the moment of anesthesia, and after that underwent ECG. In the ketoprofen group, premature atrial contractions (PACs) and ten dogs (out of 14) showed a decrease in heart rate was seen after injection of thiopental, which can be due to drug interaction (Figure 2). However, there was no arrhythmia in the other groups.

**Figure 3:**
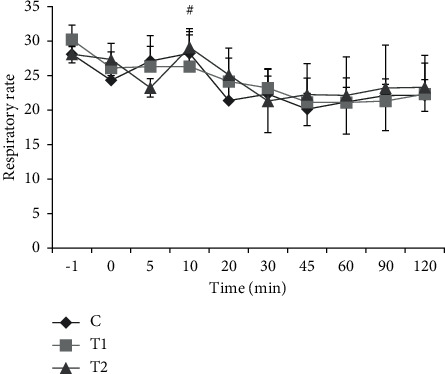
Changes in the respiratory rate in the studied groups before and after induction of anesthesia. All values are presented as the mean ± standard error of the mean (mean ± SEM). ^#^indicates a significant difference within the intragroup.

Table 2 shows the QT interval before and after thiopental injection. Before injected thiopental in all groups, there was no significant difference in QT interval, while after thiopental injection in the ketoprofen group, there was a significant increase with other groups. Analysis of the results showed that after thiopental injection up to 120 minutes later. However, the respiratory rate gradually decreased compared to before and at the time of thiopental injection, and this decrease was not significant. These changes also had no significant difference between groups. The results also showed a sudden increase in the respiratory rate at 10 minutes after injection. This change was significant in the control and T2 groups compared to before and after this time (Figure 3).

The results showed that the time of return palpebral reflex, sitting position, trying to stand, imbalanced walking, and normal walking significantly increased in the T1 group compared to the control group. However, these parameters in the T2 group had no significant difference from the control group. The recovery time was longer in the T1 group than that in the control and T2 groups. The duration of anesthesia in different groups was 115.7 ± 1.8 min in the control group, 169 ± 2.3 min in the T1 group, and 116 ± 1.9 min in the T2 group, respectively (Table 3).

**Figure 4:**
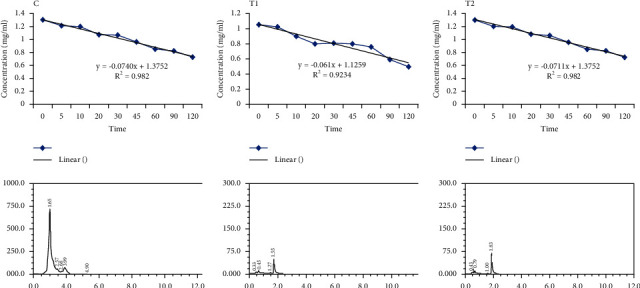
Changes in thiopental concentrations in studied groups by HPLC method.

The relationship between thiopental serum concentrations at different time intervals in control and experimental groups is shown in Figure 4. This diagram contains the hypothetical equation of diagram behavior in thiopental elimination. The *X*-coefficient in this equation represents the elimination coefficient of thiopental drug, which is the lowest in the T2 group compared to the control and T1. The following diagrams in Figure 4 show the thiopental peaks in different groups obtained from the HPLC results.

Table 4 shows changes in body weight and pharmacokinetic parameters in different groups. The body weight of dogs had no significant difference in the control and experimental groups (*P*=0.76). Thiopental elimination rate constant showed a significant decrease in the T1 group compared to other groups. The thiopental half-life significantly increased in T1 and T2 groups compared to the control group. The thiopental volume of distribution significantly increased in the T1 group compared to others. Although thiopental clearance decreased in T1 and T2 groups compared to control, it was not a significant difference (*P* > 0.05).

## 4. Discussion

It is important to understand the pharmacokinetic (PK) changes of drugs to minimize the required dose of drugs and their potential harm [25]. This feature allows physicians and veterinarians to control the concentration of the drug at the site of action under conditions such as anesthesia. It also helps achieve ideal surgical and uncomplicated recovery by titrating anesthesia according to the patient's needs [25]. Due to the weak analgesic effects of thiopental, the use of analgesic and anti-inflammatory compounds such as ketoprofen and dexamethasone could improve anesthesia and uncomplicated recovery after surgery [26].

In this study, we investigated the drug interaction of ketoprofen and dexamethasone with thiopental in healthy male dogs to improve anesthesia and uncomplicated recovery after surgery. Moreover, we evaluated the anesthetic and pharmacokinetic parameters of thiopental and changes in heart rate and respiratory rate after injection of ketoprofen and dexamethasone. The results of hematological and biochemical parameters of liver, and kidney in studied animals and physical examinations confirm the relative health of animals in this study. The liver and kidney also have normal functions. Therefore, it can be concluded that the changes in thiopental kinetics can be attributed to drug interaction conditions.

This study also showed that ketoprofen injection after induction of anesthesia by thiopental was seen in ten dogs' premature atrial contractions (PACs) and disappearance during recovery, the results that were not seen in the control and dexamethasone groups. PACs are early contractions that occur in the atrial myocardium and do not originate in the atrial sinus node (SA node) [27]. This possible mechanism is an imbalance between the production of PGI2 and thromboxane A2 due to the production of nitrous oxide from the vascular endothelium, which alters the Q-T distance in the ECG [28].

The QT interval is a dynamic physiological variable that can be affected by the velocities of both ventricular conduction and repolarization. Multiple factors have been described to affect the QT interval, such as the cardiac cycle length, autonomic nervous system activity, age, gender, circadian rhythm, plasma electrolyte concentrations, and variations in ion channels involved in cardiac repolarization [29]. Also, drug regulatory agencies have shown increasing interest in the topic because certain drugs can prolong the QT interval to a level that produces ventricular arrhythmias. In the study, QT interval in the ketoprofen group in the electrocardiographic analysis increased, and it confirmed this mechanism in the creation of the PACs. However, ketoprofen is one of the propionic acid groups of nonsteroidal anti-inflammatory drugs (NSAIDs) with analgesic and antipyretic effects. It inhibits the production of proinflammatory prostaglandin precursors by inhibiting cyclooxygenase enzymes (COX-1 and COX-2) [30].

The recovery parameters in thiopental anesthesia showed a significant increase in the group receiving ketoprofen compared with dexamethasone, expect a normal decrease in heart rate and respiration. It did not show any significant change among the controlled and treated groups compared to before. The evaluated pharmacokinetic parameters of thiopental showed a significant decrease in thiopental elimination coefficient and increased its half-life with ketoprofen. The volume of thiopental distribution in the ketoprofen group showed a significant increase compared to the control and dexamethasone groups. However, the decrease in thiopental clearance in the experimental groups was not significant compared to the control group.

To interpret these results, we first discuss selecting the doses of drugs used in this study. The recommended dose of thiopental was chosen from the study of Raeeszadeh and Rajaian [20]. The recommended dose of ketoprofen was chosen from the study of Delage et al. [31] and the dose of dexamethasone from the study of Wank et al. [32]. In these studies, the pharmacokinetic parameters of each of these drugs have been well studied. The volume of distribution depends on the cardiac output and plasma protein binding [33]. Besides, in the previous studies, ketoprofen and dexamethasone have been reported to increase the heart rate [34, 35]. Therefore, we can point to the competitive effects of thiopental in the presence of ketoprofen for binding to plasma proteins, which has increased the free form of the drug and thus increased its volume of distribution [33]. Horie et al., in a pharmacokinetic study, reported that the distribution of ketoprofen in tissues is shallow and has the highest concentration in plasma [36]. The results of our study showed an increase in thiopental half-life in the presence of ketoprofen, and this increase in half-life was due to a decrease in thiopental drug elimination. Increasing the half-life of thiopental has also reduced its clearance in drug interaction conditions. In fact, this suggests that the affinity of thiopental to bind to plasma proteins is greater than that of ketoprofen. Borgå and Borgå reported a 53% plasma protein bond for ketoprofen under exogenous conditions. They stated that the compound could cause mutations in the binding site and drug interaction conditions with other drugs [37]. Accordingly, the results of this study indicate drug interaction and thiopental binding site shift in the presence of ketoprofen. The concentration of thiopental in serum decreased at zero time, and its distribution volume was increased. Increasing the free form of thiopental in the conditions of ketoprofen drug interaction causes its distribution in other tissues, especially adipose tissue, which leads to an increase in the volume of thiopental distribution. The results of this study were in line with the study of Borgå and Borgå [37] and Raeeszadeh and Rajaian [20] on the interaction of phenylbutazone with thiopental. Significant reduction of thiopental elimination rate and increase of thiopental half-life and consequently increase of its recovery time compared with dexamethasone and control group indicate thiopental redistribution from tissues to blood in this interference [35, 38, 39]. A single dose of dexamethasone before surgery can control nausea and vomiting and, to some extent, pain after surgery [40, 41]. Also, a single perioperative dose of dexamethasone (IV) had small but statistically significant analgesic benefits [42]. It was recommended that the use of dexamethasone in surgery at doses higher than 0.1 controls the postoperative pain and reduces the use of opioid analgesics [40, 41]. Considering the pharmacokinetic and clinical changes of dexamethasone drug interaction with thiopental, there were controlled effects of this drug interaction with thiopental compared to ketoprofen. We know that increasing the recovery time in conditions of thiopental drug interaction with ketoprofen and severe weakening of the respiratory center is very common in the simultaneous use of opioids [43]. Therefore, due to the minimal drug interaction, dexamethasone and thiopental are recommended under anesthesia instead of ketoprofen.

## 5. Conclusion

Drug interaction of thiopental with ketoprofen by reducing the elimination rate and increasing the volume of distribution causes an increase in recovery time from thiopental anesthesia. However, dexamethasone at a dose of 0.2 mg/kg with insignificant changes in the pharmacokinetic parameters of thiopental and its anesthetic parameters can be used in surgery and anesthesia with thiopental. However, compartment analysis is recommended to complete the results of the thiopental pharmacokinetics parameters.

## Figures and Tables

**Table 1:** Biochemical and hematological parameters evaluated in the controlled and treated groups.

Parameters	Mean ± SEM control	Mean ± SEM T1	Mean ± SEM T2	*P* value	Normal range
W.B.C × 10^3^ (meq/l)	12.5 ± 2.2	11.33 ± 3.4	10.5 ± 2.6	0.343	6–17
R.B.C × 10^6^ (meq/l)	6.59 ± 1.98	7.49 ± 1.8	6.22 ± 1.47	0.964	4–9
HGB (g/dl)	16.9 ± 1.87	16.6 ± 2.6	17.3 ± 1.9	0.562	12–18
HCT (%)	49.3 ± 3.80	43.5 ± 2.25	50.3 ± 5.72	0.256	37–55
M.C.V (fl)	64.7 ± 2.60	65.3 ± 1.45	66.3 ± 4.65	0.363	60–72
M.C.H.C (pg)	21.4 ± 1.31	22.4 ± 1.70	23.4 ± 1.92	0.671	19.5–25.5
PLATELET × 10^3^ (meq/l)	467.6 ± 45.31	470.3 ± 26.32	480.29 ± 28.27	0.384	200–500
Neutrophil (%)	66.56 ± 10.2	63.2 ± 5.78	65 ± 9.31	0.223	60–77
Lymphocyte (%)	19.3 ± 3.12	19.5 ± 3.42	20.3 ± 7.83	0.652	12–30
Monocytes (%)	2.8 ± 1.32	3.0 ± 1.46	2.9 ± 1.15	0.423	3–10
Eosinophil (%)	0.1 ± 0.01	0.1 ± 0.32	0.1 ± 0.13	0.281	0.1–1.3
Basophil (%)	0.25 ± 0.12	0.1 ± 0.10	0.1 ± 0.47	0.572	0–0.5
AST (U/L)	27.4 ± 13.4	26.5 ± 15.2	25.34 ± 12.5	0.342	10–34
ALT (U/L)	43.35 ± 10.67	44.56 ± 15.8	41.0 ± 13.46	0.195	7–70
Urea (mg/dl)	27.16 ± 11.7	29.16 ± 8.7	26.22 ± 5.15	0.148	17–38
Creatinine (mg/dl)	0.8 ± 0.39	0.7 ± 0.47	0.9 ± 0.65	0.210	0.3–1.7
Total protein (g/l)	52.3 ± 3.7	54.3 ± 4.89	50.4 ± 4.67	0.231	50–78

**Table 2:** Changes in QT interval in the electrocardiogram in different groups.

ECG parameter	Control	T1	T2
QT interval (sec) (before injection thiopental)	0.20 ± 0.02	0.19 ± 0.01	0.21 ± 0.03
QT interval (sec) (after injection thiopental)	0.19 ± 0.03	0.29 ± 0.02 a^*∗*^	0.22 ± 0.01

Each row contains the mean ± standard error of the mean (mean ± SEM) and represents a, b, and c, respectively, compared to the control, T1, and T2 groups. ^*∗*^*P* < 0.05.

**Table 3:** Comparison of recovery time in studied groups following anesthesia induced by sodium thiopental.

Groups	Parameters (minutes)				
	Palpebral reflex return	Sitting position	Trying to stand	Imbalanced walking	Normal walking
Control	5.30 ± 0.2	16.62 ± 0.5	60.10 ± 2.9	68.71 ± 3.5	121.00 ± 2.0
T1	9.63 ± 0.2 a^*∗*^	37.40 ± 1.3 a^*∗*^	108.08 ± 9.6 a^*∗∗*^	151.71 ± 3.3 a^*∗∗*^	178.85 ± 2.5 a^*∗*^
T2	6.12 ± 0.3	21.04 ± 0.3	61.02 ± 4.0	67.85 ± 3.3	122.71 ± 2.2

Each column contains the mean ± standard error of the mean (mean ± SEM) and represents a, b, and c, respectively, compared to the control, T1, and T2 groups. ^*∗*^*P* < 0.05, ^*∗∗*^*P* < 0.01.

**Table 4:** Changes in body weight and thiopental pharmacokinetic parameters in the studied groups.

Groups	Weight (kg)	*K*_*el*(h^−1^)_	*t*_1/2(h)_	*V* *d* (L h^−1^ kg^−1^)	*Cl* (Lh^−1^)
Control	21.34 ± 0.330	0.72 ± 0.006	9.17 ± 0.074	2.81 ± 0.038	2.10 ± 0.020
T1	21.18 ± 0.23	0.61 ± 0.004 a^*∗*^	13.38 ± 0.181 a^*∗∗*^	3.41 ± 0.026 a^*∗*^	2.09 ± 0.019
T2	21.23 ± 0.24	0.71 ± 0.005 b^*∗*^	9.24 ± 0.23 b^*∗∗*^	2.74 ± 0.021 b^*∗∗*^	2.02 ± 0.024

Each column contains the mean ± standard error of the mean (mean ± SEM) and represents a, b, and c, respectively, compared to the control, T1, and T2 groups. ^*∗*^*P* < 0.05, ^*∗∗*^*P* < 0.01. *k*_*el*_: elimination rate constant, *t*_1/2_: half-life, *V*  *d*: volume of distribution, Cl: clearance.

## Data Availability

The datasets used and/or analyzed during the current study are available from the corresponding author on reasonable request.

## Additional Points

*Highlight*. (i) Unlike dexamethasone, ketoprofen injection alters pharmacokinetic parameters such as elimination rate, the volume of distribution, and half-life of thiopental in dog anesthesia. (ii) Dexamethasone at a dose of 0.2 mg/kg causes insignificant changes in the pharmacokinetics of thiopental and its anesthetic parameters. (iii) In pharmacokinetics drug interaction, differences in plasma protein binding of ketoprofen and dexamethasone were most important. (iv) Anesthesia is one of the situations in which drug interaction shows their effects. (v) Safe and effective combinations of several agents are needed to provide optimal anesthesia.
